# Transcriptomic Characterization of *Miscanthus sacchariflorus* × *M. lutarioriparius* and Its Implications for Energy Crop Development in the Semiarid Mine Area

**DOI:** 10.3390/plants11121568

**Published:** 2022-06-14

**Authors:** Hui Feng, Cong Lin, Wei Liu, Liang Xiao, Xuhong Zhao, Lifang Kang, Xia Liu, Tao Sang, Zili Yi, Juan Yan, Hongmei Huang

**Affiliations:** 1 College of Bioscience and Biotechnology, Hunan Agricultural University, Changsha 410128, China; fenghui@bzpt.edu.cn (H.F.); xiaoliang@hunau.edu.cn (L.X.); yizili@hunau.net (Z.Y.); 2 Binzhou Polytechnic College, Binzhou 256603, China; 3 Key Laboratory of Plant Resources and Beijing Botanical Garden, Institute of Botany, Chinese Academy of Sciences, Beijing 100093, China; lincong@ibcas.ac.cn (C.L.); liuw@ibcas.ac.cn (W.L.); zhaoxh17@ibcas.ac.cn (X.Z.); kanglf@ibcas.ac.cn (L.K.); sang@ibcas.ac.cn (T.S.); 4 CAS Key Laboratory of Plant Germplasm Enhancement and Specialty Agriculture, Wuhan Botanical Garden, Chinese Academy of Sciences, Wuhan 430074, China; liuxia@wbgcas.cn

**Keywords:** *Miscanthus sacchariflorus*, *Miscanthus lutarioriparius*, transcriptome analysis, metabolic pathways

## Abstract

*Miscanthus* interspecific hybrids have been proved to have better adaptability in marginal lands than their parents. *Miscanthus sacchariflorus* and *Miscanthus lutarioriparius* were used as the parents to develop hybrids. We performed the transcriptome for 110 F1 hybrids of *Miscanthus sacchariflorus × Miscanthus lutarioriparius* and their parents that had been established on the Loess Plateau mine area, to estimate the population’s genetic expression variation, and illuminate the adaptive mechanism of the F1 population. The result speculated that the F1 population has mainly inherited the stress response metabolic pathway of its female parent (*M. sacchariflorus*), which may be responsible for its higher environmental adaptability and biomass yield compared with male parents. Based on PopART, we assembled a leaf reference transcriptome for *M. sacchariflorus* (LRTMS) and obtained 8116 high-quality transcripts. When we analyze the differential expression of genes between F1 population and parent, 39 and 56 differentially expressed genes were screened out in the female parent and male parent, respectively. The enrichment analysis showed that pathways of carbohydrate metabolism, lipid metabolism, biosynthesis of secondary metabolites and circadian rhythm-plant played a key role in resisting the harsh environment. The carbohydrate metabolism and lipid metabolism were also significantly enriched, and the synthesis of these substances facilitated the yield. The results provided an insight into breeding *Miscanthus* hybrids more suited to the harsh environment of the Loess Plateau.

## 1. Introduction

In terms of the increase in demand for renewable energy and environmental improvement, developing perennial bioenergy plants on marginal land provides a feasible solution. The Loess Plateau has more than 60 Mha of marginal land, and covers a semiarid to semi-humid transitional zone. If people plant plentiful bioenergy crops in these regions, this would have positive environmental impacts with little carbon debt [1,2,3].

*Miscanthus* is considered as a C4 perennial grass with high water use efficiency and adaptability to harsh conditions, which have played a vital role in developing this bioeconomy, along with its ecological functions such as soil restoration [4,5]. *M. lutarioriparius* and *M. sacchariflorus* are species belonging to Poaceae, *Miscanthus* Anderson. *M. sacchariflorus* has a high level of genetic diversity and adaptation. Literature has also suggested that the two species had been transplanted on the Loess Plateau of northwestern China, which demonstrated that they had great potential to serve as suitable progenitors in perennial energy crop breeding programs [6,7,8]. Compared to *M. sacchariflorus*, *M. lutarioriparius* has lower heritability of traits such as cold and drought tolerance, and lower establishment rates in the Loess Plateau. On the other hand, higher photosynthetic and water use efficiency led to better biomass yield in *M. lutarioriparius* than *M. sacchariflorus* in the Loess Plateau [6,9,10,11,12].

Heterosis/hybrid vigor is a biological phenomenon which can produce offspring with superior characteristics, such as biomass yield, stature and stress management, than either parent. It can produce better cultivars if the biomass heterosis and the stress tolerance heterosis are combined, which has already been investigated by some researchers in the *Miscanthus* species [13,14,15,16]. The F1 population resulting from a cross between *M. sacchariflorus* and *M. lutarioriparius* was developed [15,16], which had significant heterosis/hybrid vigor and performed well in the marginal soil in Liuyang City, Hunan Province. In order to select fine individuals from the F1 population with outstanding characteristics, such as high biomass yield and strong stress resistance, 110 hybrids together with their parents were transplanted at Xiaoyi in Shanxi Province on the Loess Plateau of China.

Given the improvement of high next-generation sequencing, RNA-Seq has been used for the comparative analyses of gene expression, and applied to study systems without a reference genome sequence. The mature leaves in the growing period of “*M. sacchariflorus × M. lutarioriparius*” F1 population were cut in order to study and analyze the differences in adaptive gene expression between hybrids and their parents in the Loess Plateau at the transcriptome. The expression and transcriptional regulation of adaptable genes of the hybrid comparing *M. sacchariflorus* and *M. lutarioriparius,* transplanted to the Loess Plateau, and how this changed according to the marginal land is not reported. Furthermore, the analysis of transcriptome characteristics can give a new perspective to the hybrids’ adaptation mechanisms under different stress conditions.

We carried out the population genetic expression variation on the Loess Plateau to understand the genetic basis of the higher biomass and adaptation in the hybrid *Miscanthus*. Studies on the transcriptome of Miscanthus hybrids are concentrated on *M. sinensis* × *M. sacchariflorus*, but research on *M. sacchariflorus* × *M. lutarioriparius* has rarely been reported [17,18]. By analyzing the genome-wide comparative transcriptome, DEGs, gene expression patterns, and the biological pathways that mediate adaptation and biomass vigor in hybrid *Miscanthus* were identified. The result gave a better understanding of the advantages in growth, stress resistance and adaptability in heterosis. In addition, in the future these data resources will be important to find candidate genes for biomass vigor in hybrid *Miscanthus*.

## 2. Results

### 2.1. De Novo Assembly of Reference Sequence of Leaf Transcripts of M. sacchariflorus

RNA sequences of *M. sacchariflorus* obtained 2.127 × 10^10^ 100-bp paired-end reads after data filtering and quality control. Based on RNA-seq, we developed a PopART workflow to assemble leaf reference transcripts of *M. sacchariflorus*. The Scaffolds formed an assembly group by combining Scaffolds and CAP3, which was obtained from SOAP-Trans, resulting in 8116 sequences, with more than 50 amino acid residues in each sequence. The combination of identity > 70% and overlap > 60% was selected as the clustering threshold, and it was speculated that the number of the transcriptome was close to that of the leaf. The comparison of sequence identity with BOSZ was reasonable.

BLASTP was used to compare these clusters in the BOSZ protein database and obtained 8116 sequences with the best results (Table 1).

### 2.2. Analysis of Gene Expression in Hybrid Population

**Figure 1:**
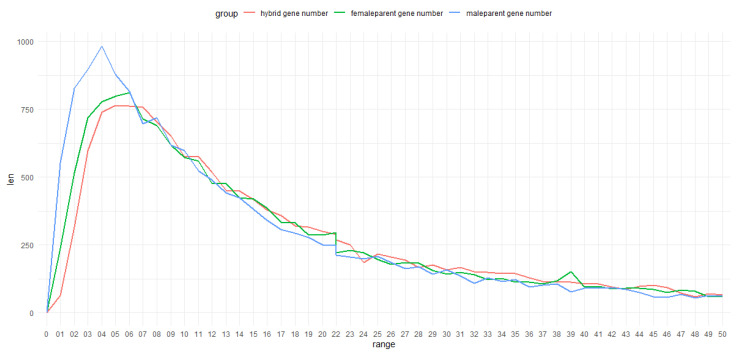
The FPKM Distribution Comparing F1 Population and Parents. The red line demonstrates the range of the FPKM distribution between 0 and 50 in the hybrid. The green line demonstrates the range in the FPKM distribution between 0 and 50 in *M*. *sacchariflorus.* The blue line demonstrates the range of the FPKM distribution between 0 and 50 in *M*. *lutarioriparius*.

The distribution and quantity of Ep value in the F1 population were calculated, and compared with the FPKM value of two parents. It was found that the distribution of the Ep value of hybrid progenies and their parents was roughly the same, and their correlation coefficient was 0.96 and 0.95, respectively (Figure 1). According to the FPKM distribution of the F1 population and its two parents, the EP value of the F1 population was higher than that of its parents, and the FPKM value of *M. sacchariflorus* was higher than that of *M. lutarioriparius*.

These results indicates that the gene response of F1 population was more active than its parents in the Loess Plateau, which suggests that the F1 population had better adaptability to the marginal land environment than its parents, and the female parent (*M.* *sacchariflorus*) was more adaptable than the male parent (*M.* *lutarioriparius*).

### 2.3. Screening of Differential Genes and Gene Expression Analysis

**Figure 2:**
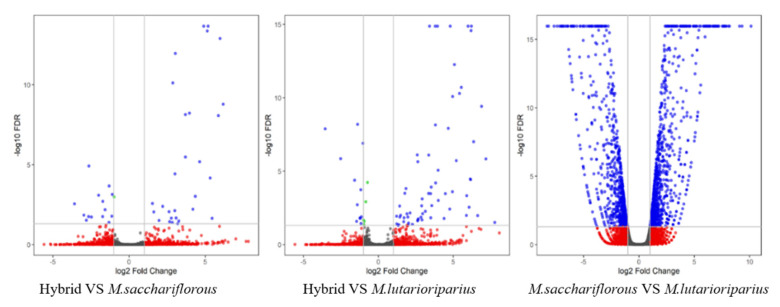
The Volcano Plots of Differential Expression Genes. Blue dots on the right represent up-regulated DEGs and blue dots on the left represent down-regulated DEGs.

Screened by EBSeq, there were 39 DEGs inDEG1, which included 12 down-regulated genes and 27 up-regulated genes; there were 56DEGs in DEG2, which included 11 downregulated genes and 45 up-regulated genes; there were 1614 DEGs in DEG3, which included 734 down-regulated and 880 up-regulated genes (Figure 2).

**Figure 3:**
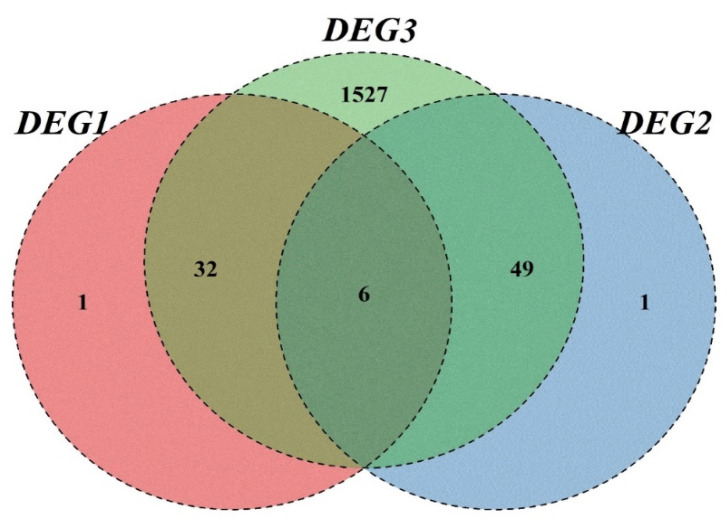
The Analysis of Differential Expression Genes in Three Differential Groups. The sum of the numbers in each large circle represents the total number of DEGs between combinations. The overlapping part of the circles represents DEGs for the treatment combinations. “DEG1”: number of DEGs between *M. sacchariflorus* and *M. lutarioriparius*; “DEG2”: number of DEGs between hybrids and *M. lutarioriparius*; “DEG3”: number of DEGs between hybrids and *M. sacchariflorus*.

**Figure 4:**
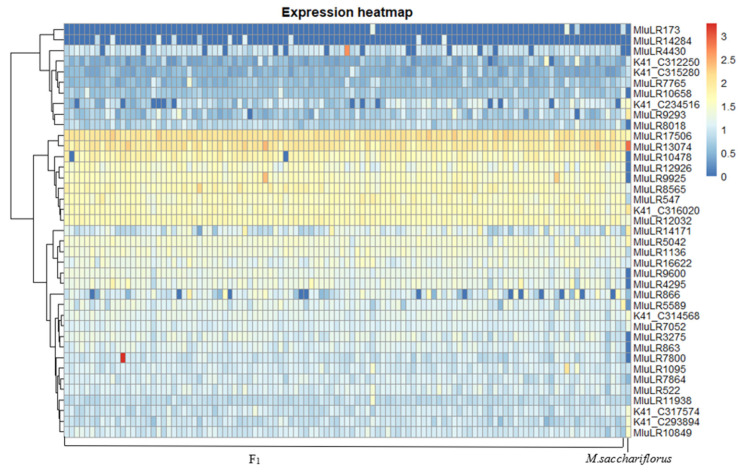
Clustering of Expression of Differential Expression Genes between F1 vs. *M. sacchariflorus*.

**Figure 5:**
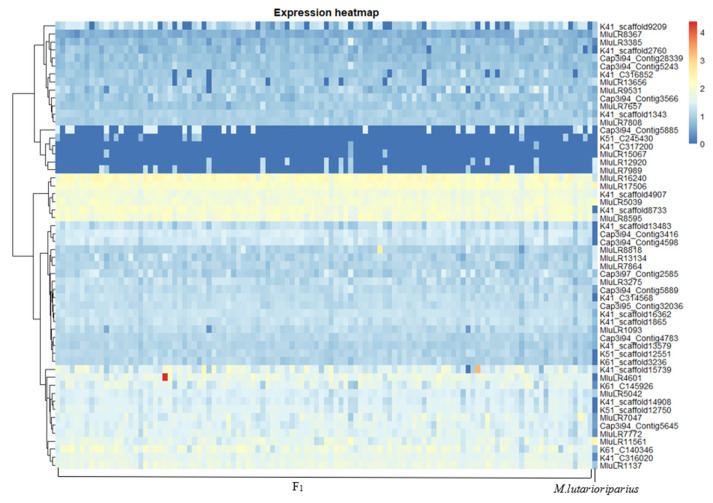
Clustering of Expression of Differential Expression Genes between F1 vs. Male parent.

Analyzing the differential genes, we found that DEG3 contained most of DEG2 and DEG1, and there were 33 specific response genes (RRDEG1) and 50 specific response genes (RRDEG2) in the F1 population relative to female parent and male parent, respectively (Figure 3). According to the hierarchical cluster analysis of Spearman rank correlation, the differentially expressed genes in the F1 population relative to parents could be clustered into five groups (Figure 4 and Figure 5).

The gene expression multiples (DEG (log_2_ (FC)) of different genes (Table S2) analyzed, and some discoveries, were found as following:

(1) The down-regulated genes of RRDEG1 (log_2_ (FC)) were up-regulated in DEG3 (log_2_ (FC)), and the up-regulated genes of RRDEG1 (log_2_ (FC)) were down-regulated in DEG3 (log_2_ (FC)), but the values of RRDEG1 (log_2_ (FC)) were all mostly lower than those of DEG 3 (log_2_ (FC)).

(2) The regulation of DEG2 (log_2_(FC) was consistent with that of DEG3 (log_2_(FC), but the value of DEG2 (log_2_(FC) was lower than that of DEG3 (log_2_(FC).

The results suggested that some traits controlled by DEGs of the F1 population generation were stronger than that of *M. lutarioriparius*, but weaker than that of *M. sacchariflorus*.

### 2.4. GO Functional Annotation and Enrichment Analysis of Differential Genes

#### 2.4.1. GO Functional Annotation and Enrichment Analysis of Differential Gene

**Figure 6:**
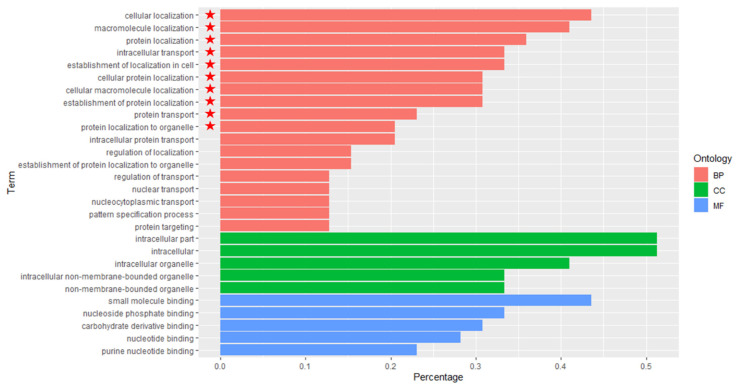
GO Enrichment of Expression of Differential Expression Genes between F1 vs. Female parent. The statistically significant difference of GO enrichment of expression of differential expression genes is marked as 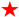.

DEG1 were enriched and annotated with GO (Figure 6), and 185 GO terms were enriched and associated with the biological process; of these, 10 terms were statistically significantly different.

It was found that none of the GO terms were statistically significantly different, which was associated with cellular compartment and molecular function.

There are five kinds of functions in the biological process: localization, biological process regulation, metabolic process, cell metabolic process, and a single a biological process (Table S3).

Comparing the GO enrichment and annotation of DEG3 with DEG1, the function of phosphorus and phosphate metabolism regulation were lacking.

**Figure 7:**
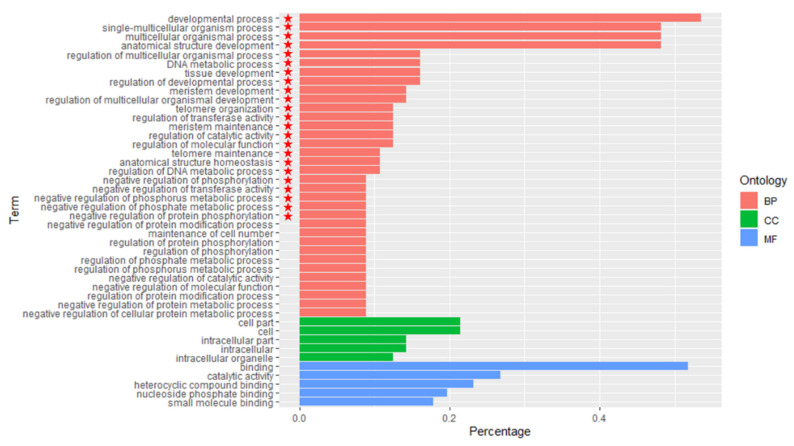
GO Enrichment of Expression of Differential Expression Genes between F1 vs. Male parent. The statistically significant difference of GO enrichment of expression of differential expression genes is marked with 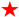.

Meanwhile, DEG2 were enriched and annotated with GO (Figure 7). We found that none of the GO terms associated with the biological process, cellular compartment and molecular function were statistically significantly different.

There are six kinds of functions in the biological process, including biological process regulation, etc. Comparing the GO enrichment and functions annotation of DEG2 with DEG1, the function of phosphorus, phosphate and phosphate metabolism, enzyme activity and protein phosphorylation were added, while the functions of cell location regulation, small molecule metabolism and cellular amine were lacking (Table S3).

To sum up, three groups of differentially expressed genes, GO annotated and enriched, were significantly different in the biological process, but there were no significant differences in cell composition and molecular function.

The enrichment and annotation functions of DEG2 were the greatest, which were similar in type and quantity to DEG1, including regulation of phosphate, phosphate metabolism, regulation of enzyme activity and regulation of protein modification process. In contrast, the enrichment and functions annotation of DEG2 were the least, only including location regulation, and phosphorus and nucleoside diphosphate metabolism. The result indicated that some traits of F1 population associated with the biological process were more similar to the female parent (*M. sacchariflorus*) than to the male parent (*M. lutarioriparius*).

#### 2.4.2. Functions Annotation and Enrichment Analysis of KEGG Metabolic Pathway

In DEG3, we obtained 49 significantly different metabolic pathways from a total of 109 KEGG metabolic pathways, which distributed five categories of A class pathway: metabolism, genetic information, environmental information processing, cell process and biological system. There were 33 metabolic pathways of B class, including sugar metabolism, lipid metabolism, amino acid metabolism and biosynthesis of secondary metabolites. These metabolic pathways account for 82.5% of the total (Table S3).

The results suggest that the differences in agronomic traits between female and male parent might be related to carbohydrate metabolism and lipid metabolism, while the differences in adaptability in Loess Plateau mining areas might be related to amino acid metabolism and biosynthesis of secondary metabolites. Moreover, the MAPK signaling pathway in the environmental information processing pathway, plant and plant hormone signal transduction, peroxisome in cell process, plant circadian rhythm and plant pathogen interaction pathway in the biological system are all reasons for the different adaptability between female and male parents.

**Figure 8:**
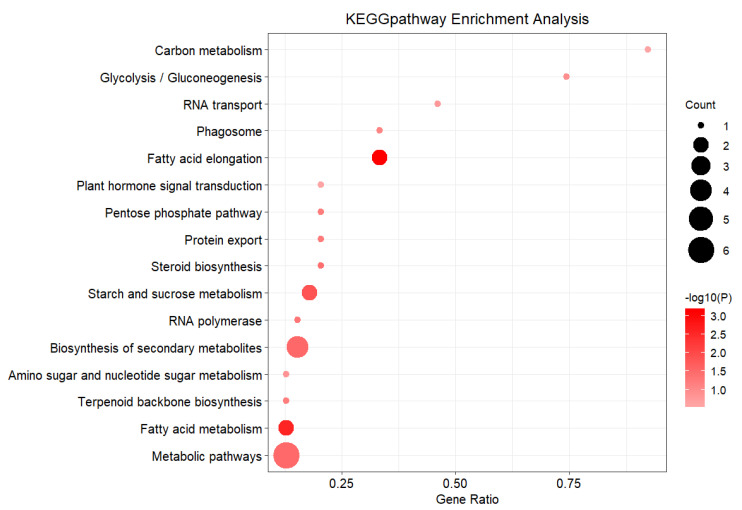
KEGG Enrichment of Expression of Differential Expression Genes between F1 vs. Female parent.

There were seven significantly different metabolic pathways in a total of 16 KEGG metabolic pathways (Figure 8), enriched and annotated in DEG1. (Table S3). Among significantly different metabolic pathways, overall there are six, of which the glucose metabolism pathway and lipid metabolism pathway account for 85.7%.

It was reported that there were small differences in agronomic traits between the F1 population and the female parent (*M. sacchariflorus*), which might be related to the metabolism of starch and sucrose in the C class of glucose metabolism pathway, or fatty acid extension and steroid biosynthesis of the C class of lipid metabolism pathway. The differences in adaptability in the experimental areas might be related to the biosynthesis of secondary metabolites and metabolic pathway of the C class of the overall pathway, or the RNA polymerase of the third layer of the genetic information processing pathway.

**Figure 9:**
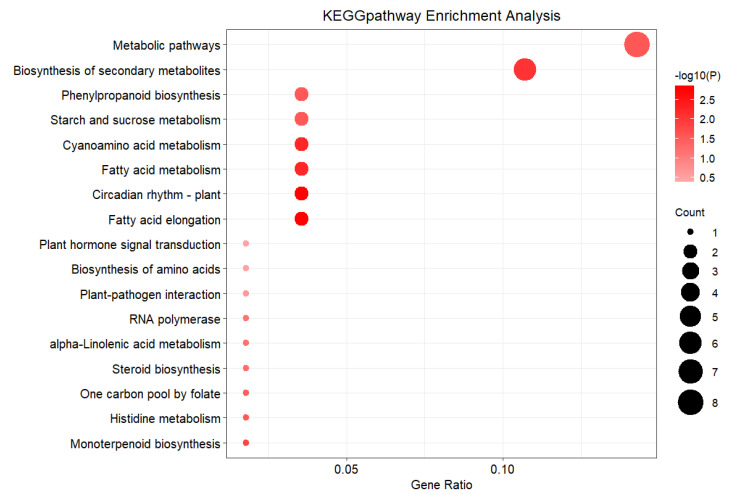
KEGG Enrichment of Expression of Differential Expression Genes between F1 vs. Male parent.

There were 11 significantly different metabolic pathways which were DEG2 enriched and annotated (Figure 9), in which the quantity and the up-down regulation condition of genes were different (Table S3).

There were eight significantly different metabolic pathways of the B class, accounting for 90.9% of all pathways. This is relevant for the glucose metabolism pathway, lipid metabolism, terpenoids and poly-ketones metabolism pathway, etc. This refers to the differences in agronomic traits between the F1 population and the male parent, possibly because of the metabolism of starch and sucrose in the C class of glucose metabolism pathway, fatty acid extension of the lipid metabolism pathway, metabolism of coenzymes and metabolic vitamins in the C class of coenzyme and vitamin metabolism pathway, and the differences in adaptability to the experimental areas, possibly because of the biosynthesis of amino acid metabolism, biosynthesis in the secondary metabolites, and plant circadian metabolic processes in the biological system pathway.

In conclusion, there was no significant difference in the metabolic pathway of stress resistance between the F1 population and *M. sacchariflorus*, while there were some differences in starch and sucrose metabolism in the carbohydrate synthesis pathway and fatty acid prolongation pathway, and steroid biosynthesis in the lipid metabolism pathway, when comparing the F1 population and *M. sacchariflorus*.

The results showed that there were many differences in the metabolic pathways of stress resistance between the F1 population and *M. lutarioriparius*. All of the DEGs were enriched in the amino acid metabolism and the biosynthesis of secondary metabolites in the metabolic pathway, the plant circadian rhythm in the biological system pathway, starch and sucrose metabolism in the carbohydrate synthesis metabolism pathway, and fatty acid prolongation in the lipid metabolism pathway.

## 3. Discussion

Stress induces the expression of related genes in plants, which leads to the synthesis of small signal molecules through the relevant metabolic pathways, so that plants can make adaptive response to the stress environment.

Intraspecific hybrids usually show concomitant enhancement in both growth and stress tolerance through a regulatory network [19]. In recent years, transcriptome sequencing technology has been used to study the gene regulation of *Miscanthus* plants under a stress environment. A large number of stress response genes have been identified and some response mechanisms have been elaborated [20,21,22,23].

The transcriptome level investigation for *M. sacchariflorus*, *M. lutarioriparius* and their F1 populations under stress conditions in the Loess Plateau was explored for the first time as part of this study.

### 3.1. Genetic Basis at Transcriptional Level of Heterosis for Miscanthus

Heterosis utilization is an effective way to create new plant germplasm. The *Miscanthus* hybrid plants compared with their parents have greater advantages in stress resistance, ecological adaptability and biomass yield. In this experiment, the distant geographical and interspecific discrepancy between the two parents resulted in a better heterosis in the F1 population. Compared with their parents in the barren land of Liuyang, Hunan Province, the F1 population showed better adaptability to drought. Moreover, *M. sacchariflorus* has performed better than *M. lutarioriparius* in the mining area of Luliang in Shanxi Province, which is close to the native area of *M. sacchariflorus*. The F1 population has a similar overwintering rate, planting rate, higher survival rate, and higher biomass yield compared with *M. sacchariflorus* (result to be published).

The quantity of DEG2 (56) was lower than that of DEG1 (39). DEG2 was mainly enriched and annotated in 4 KEGG pathways, including starch and sucrose metabolism, fatty acid elongation, steroid biosynthesis and RNA polymerase. DEG1 was mainly enriched and annotated in 7 KEGG pathways, including starch and sucrose metabolism, fatty acid elongation, histidine metabolism, cyano-amino acid metabolism, one carbon pool by folate, monoterpenoid biosynthesis, phenylpropanoid biosynthesis and circadian rhythm. The number of DEGs showed that the gene expression of the F1 population was more similar to the female parent (*M. sacchariflorus*) than to the male parent. From this it can be deducted that the female parent (*M. sacchariflorus*) contributed more and achieved more genetic advantage in the F1 population in terms of high adaptability. Some studies have found that the difference in genes between F1 population and their parents are significantly enriched in the carbohydrate metabolism pathway [24,25,26], which indicates that the metabolism of carbohydrate, energy, and amino acids is closely related to plant growth and development. In this study, the DEGs in the comparison between F1 population parents were also enriched in starch and sucrose metabolism and the fatty acid prolongation metabolism pathway, which could provide material and energy for growth and development.

In conclusion, the transcriptome analysis of the F1 population and its parents indicted that the hybrid population inherited its superior stress resistance from the female parent (*M. sacchariflorus*). Furthermore, the significant differences in starch and sucrose metabolism and the fatty acids elongation metabolism might result in F1 population’s higher adaptability and biomass yield in the Loess plateau environment than that of its parents.

### 3.2. Metabolic Pathways in F1 Population and Parents in Response to Combined Stress

Metabolic pathway of significant enrichment of genes in the F1 population compared with their parents, including biosynthesis of secondary metabolites, fatty acid metabolism, carbohydrate metabolism, RNA polymerase and amino acid metabolism, and cofactors and vitamins metabolism, were identified as relevant to the ability of plants to tolerate stress in a large number of studies.

For instance, some organic metabolites (such as free amino acids, soluble sugars, etc.) were synthesized and accumulated by plants through carbohydrate metabolism to maintain cell osmotic pressure [27,28,29] to tolerate stress conditions. The fatty acid extension pathway of leaves is activated under drought stress, and cuticle deposition and cell elongation occur simultaneously. The accumulation of wax in the cuticle of plants can prevent chlorophyll leaching, reduce water retention on leaf surface, protect plants from enhanced ultraviolet radiation, and protect plants against air pollution and weathering during growth in order to overcome drought conditions. However, cells begin to stop elongation when a large amount of wax is deposited [30,31,32,33].

In this experiment, we found wax powder on the leaf sheath and stem surface of the hybrid, and at the transcriptome level the DEGs were significantly enriched in the fatty acid extension pathway, which suggested that cuticle wax biosynthesis of the hybrid may be one reason for better resistance to the harsh environment than that of its parents.

Furthermore, secondary metabolites (including flavonoids, lignin, coumarin, etc.) are synthesized and accumulated through flavonoid biosynthesis and the phenyl-propionic acid biosynthesis pathway [34], which increased the cell wall hardness and formed the vascular tissue, protective tissue and mechanical tissue, which can enhance drought resistance and aluminum stress [35,36].Thus the biomass yield of plants and their adaptability to adversity can be improved by circadian rhythm regulation [37].

These metabolic pathways lead to the change of chemical components in functional organs of plants and improve the adaptability of plants to adversity, which reveal the reasons why the F1 population group can be more adaptive to the harsh environment in the Loess Plateau than its parents.

### 3.3. Relationship between Cell Wall Changes and Adaptability of Miscanthus in Loess Plateau

Plant cell walls have highly complex and dynamic structures, which are composed of polysaccharides, structural proteins and other polymers, and not only provide mechanical support, but also support plant growth. These structural components play an active defense role in biological and abiotic stresses in response to various environments. Cell walls are usually divided into primary wall and secondary walls. The primary cell wall is the dynamic structure supporting the growth of plant cells, and its expansion is the basis of plant morphology. The secondary cell wall is deposited after the cell expansion, and finally provides the plant support and hardness. The secondary wall provides most of the plant biomass, mainly composed of cellulose, hemicellulose and lignin. Some diverse genotypes in *Miscanthus* species significantly reduced cell wall and cellulose content when they suffered drought stress [21,38]. Saccharification efficiency is often negatively correlated to cell wall content [38]. Carbohydrate metabolism accelerates cellulose conversion into permeable substance (such as soluble sugar, proline, etc.) to maintain cell osmotic pressure which leads to improved drought resistance capability. Compared with their parents, the differential genes between the parents and the hybrids were enriched in the carbohydrate metabolism pathway, and the differential gene expression multiple (DEG (log_2_ (FC)) was up-regulated, which may be one of the main reasons why the hybrids were superior to their parents in stress resistance. The phenylalanine metabolism pathway is related to drought, which can synthesize lignin. The lignin, which is deposited in the secondary cell wall, has the function of water transport and enhancing cell compression and tension [39,40], which can subsequently improve drought tolerance. However, there is insufficient evidence to establish that a mechanistic relationship between drought stress and lignin exists. It has been reported that lignin content increased under drought stress [41,42], whereas in another study it was found that drought had little effect on lignin content of the cell wall [38].

The *M. sinensis*, *M. sacchariflorus* and *M. lutarioriparius* were cultivated and evaluated in the Yellow River Delta, and *M. lutarioriparius* had the highest content of lignin [43]. In another study it was reported that the drought tolerant genotypes accumulated more lignin than the sensitive ones under drought stress [38]. Compared with the male parent (*M. lutarioriparius*), the phenylalanine metabolic pathway of the hybrid population was significantly enriched, and the one carbon pool by folate associated with the phenylalanine metabolism pathway was also significantly enriched and up-regulated.

The results suggested that the phenylalanine metabolic pathway of the hybrid was more active than that of *M. lutarioriparius* under stress, which resulted in the production of more lignin and high resistance to drought stress.

### 3.4. Relationship between Circadian Rhythm and Adaptability of Miscanthus in the Loess Plateau

Circadian rhythm refers to the movement of life activities in a certain period of time [44], which is related to plant carbon metabolism, stress adaptation, hormonal signals, light morphogenesis and defense signals. It is influenced by environmental signals (such as light, temperature and nutritional status), and controls the biological processes of plants through transcription and post transcriptional regulation of many output processes [45,46,47,48,49]. The changes of gene expression in circadian rhythm have effects on photosynthesis, carbohydrate and accumulation of chlorophyll, starch and sugar, which can improve biomass yield and adaptability to stress [37,45,50]. The difference between the original habitat and the ecological environment in the Loess Plateau led to the enrichment of the differential genes in the female parent (*M. sacchariflorus*) and the F1 population in the plant circadian rhythm, which may be the reason for better adaptability to stress of the F1 population than that of the male parent (*M. lutarioriparius*). The DEGs between F1 population and the male parent were also enriched in histidine metabolism, starch and sucrose metabolism, and both showed upregulation, which may be the positive effect of circadian rhythm on photosynthesis and distribution of organic products in the F1 population.

In conclusion, the F1 population inherited the partial characteristics of metabolism, including genetic information processing, environmental information processing, cellular process and biological system metabolic pathway. The metabolic pathway including carbohydrate and sucrose, fatty acid elongation, phenylpropanoid biosynthesis and plant circadian rhythm may have contributed towards better adaptability under harsh conditions in hybrids than male parent (*M. lutarioriparius*). Furthermore, this would also be the reason for the higher biomass yield compared with the female parent (*M. sacchariflorus*).

## 4. Materials and Methods

### 4.1. Establishment of Hybrid Population and Collection of Samples

**Figure 10:**
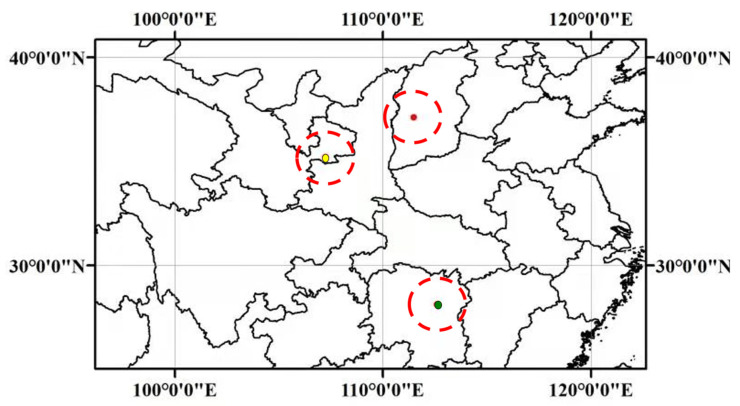
Locations of Pingliang City in Gansu Province (PG, red pot, 37.12° N, 111.45° E), Changsha City in Hunan Province (CH, green pot, 28.12° N, 112.59° E) and Lvliang City in Shanxi Province (LS, yellow pot, 35.19° N, 107.23° E) of China.

The hybrid seeds were bred naturally in Hunan Agricultural University from *M. sacchariflorus* and *M. lutarioriparius. M. sacchariflorus* (NO. A0405), whose native habitat is Yaodian, Jingchuan Town, Pingliang City, Gansu Province (PG), China, was used as female parent. *M. lutarioriparius* (NO. A0107), whose native habitat is Hunan Province (CH) of China, was used as male parent. F1 population from a manmade interspecific cross between *M. sacchariflorus* and *M. lutarioriparius* was obtained in Changsha City, Hunan Province (CH), and then transplanted to mine recovery area in Yangquanqu village, Xiaoyi County, Lvliang City, Shanxi Province (LS). The temperature in this region varies greatly, the average annual rainfall was 460.7 mm and the annual average evaporation was 1866.9 mm. It is an ideal area for screening hybrid varieties with high stress resistance due to cold, drought, barren soil and heavy metal stress. The specific locations of Pingliang, Changsha and Luliang are marked in Figure 10.

In May 2018, F1 population and their parents were bred with cloned rhizomes and 10 randomly clonal rhizomes to Xiaoyi. On the basis of the random design, cloned rhizomes of each F1 hybrid were transported to the plots of 1 m × 1 m in the experiment field. Plots were irrigated once on the first day after planting, and no longer irrigated.

The fourth mature leaf from the top of each individual plant, including 110 hybrid descendants and their parents, were cut and placed into liquid nitrogen immediately, then stored for RNA extraction, at noon from 2 to 4 September 2018.

### 4.2. RNA-Seq Processing and Assembly of Reference Transcriptome of M. sacchariflorus

Split the total RNA of each leaf sample with Trizol reagent and purify (Invitrogen, Waltham, MA, USA) with the RNeasy Mini kit (Qiagen, Hilden, Germany) respectively. Utilize the isolated mRNA to construct the 100 bp paired-end library NEBNext mRNA Library Prep Reagent Set for Illumina (NEB) and perform the sequencing on Illumina HiSeq 2500.

In order to control the quality of raw reads, FASTQC [51] and the FASTX-Toolkit [52] were used to filter and trim the RNA-Seq data. Sequentially, the first 9 bases were identified and trimmed for unstable sequence content. The data were trimmed for all samples, and generated a total of 518.12 Gb of sequence (Table S1). A pipeline of RNA-seq to assemble reference transcriptome of *M. sacchariflorus* was developed by referring to the method in [53]. Including female parent of hybrid, the reference transcriptome was formed from 6 individuals of *M. sacchariflorus*.

Sequentially, the group was performed using SOAP denovo-Trans [54], with parameters set “max_rd_len = 80, rd_len_cutof = 80, avg_ins = 200, reverse_seq = 0, asm_flags = 3 map_len = 32”, and three *kmer* values, including 41, 51, and 61, were used in each assembly group. The resulting scaffolds were pooled and merged. Some missing sequences were filled with GapClose [55], and the overlap length set at 25. The results from GapClose were merged and extended by CAP3 [56] program, and the value of *p* was set at six conditions: 99, 98, 97, 96, 95 and 94. The results of gapcloser and cap3 were combined. The EMBOSS package was utilized to predict the open reading frames (ORFs) of all these sequences, which combined the results of GapColser and CAP_3_, and the scaffold were retained, which sequences with ORFs larger than 150 bp and making up at least 30% [57]. The protein cluster analysis of Bosz protein database composed of four species of the grass family (including *B. distachyon*, *O. sativa*, *S. bicolor*, and *Z. mays*) was carried out by the program CD-HIT-2D.

The clustered sequences were sought in *M. sacchariflorus* and the BOSZ protein database using reciprocal BLASTP search [58]. The transcripts of *M. sacchariflorus*, which is the best reciprocal BLASTP combination pairs, were selected as reference sequences.

### 4.3. Expression Analysis of Hybrid Population

The *M. lutarioriparius* [59] and *M. sacchariflorus* reference transcript sequences were combined to form a new comprehensive reference transcript. The trimmed reads were mapped to the Bowtie-build index of the reference transcript, and the default setting was used in TopHat [60,61]. Cufflinks (v2.2.1) was used to describe the expression abundance of each gene. In order to select the extremum, we added the FPKM value of each gene (the expected number of fragments per million fragments per thousand base transcripts), and then transformed into log_2_ [53]. Next, quartile analysis is performed on the converted values to filter out values larger than 1.5 times the quartile range. Finally, 8116 transcripts were obtained by quartile analysis.

Ep was denoted as population gene expression, which was calculated as the average of FPKM values of the individuals sampled from the population, with the formula as follows:

$$E_{p}=\frac{\sum_{i=1}^{n}E_{i}}{n}.$$

where *n* represents the number of individuals sampled from the population and *E_i_* represents the FPKM of a given gene of the *i*th individual in the population [59].

### 4.4. Analysis and Classification of Differential Genes

The empirical Bayes hierarchical model (EBSeq) [62] was applied to screen the different genes (DEGs) by analyzing the gene expression. The DEG1 and DEG2 were screened by comparing the hybrids with the *M. sacchariflorus* and *M. lutarioriparius,* respectively. TheDEG3 were screened by comparing the *M. sacchariflorus* against the *M. lutarioriparius*.

The false discovery rate (FDR) of each transcript was calculated and the fold change (FC) was estimated. If the FDR of gene expression between the two groups is less than 0.05 and log_2_(FC) ≥ 1 or ≤ −1, it is considered that there is significant difference between the two groups. At the same time, we regarded Log_2_ (FC) ≥ 0 as upregulation and log_2_ (FC) ≤ 0 as downregulation.

The DEGs existing in DEG1 and DEG2 simultaneously were defined as shared responsive genes (SRDEG), the others defined as specific responsive genes (RRDEG).

### 4.5. Function Annotation and Metabolic Pathway Enrichment Analysis of Differential Genes

Agrigo [63] was used to classify the differentially expressed genes with *p* value less than 0.01 for GO function classification. KOBAS3.0 was used for annotation and enrichment of KEGG pathway in *Arabidopsis thaliana* database [64]. FDR value less than 0.05 was considered to be statistically significantly different. KOBAS3.0 was used to annotate and enrich the metabolic pathways associated with DEGs among male and female parents in F1 population. The metabolic pathways with significant difference were analyzed.

## 5. Conclusions

By transcriptome analyzing, the reason why the F1 population was able to adapt to various stresses in the Loess Plateau and had higher biomass yield than their parents were concluded, which are as follows: (1) the DEGs between the F1 population and its female parent (*M. sacchariflorus*) annotated the functions of the carbohydrate metabolism, lipid metabolism, biosynthesis of secondary metabolites and circadian rhythm-plant, which may lead it to be more adaptable for the harsh environment of the Loess Plateau than the male parent (*M. lutarioriparius*); (2) the DEGs between the F1 population and its male parent (*M. lutarioriparius*) annotated the functions of the carbohydrate metabolism and lipid metabolism response to the Loess Plateau, which may lead to be more higher biomass yield of the hybrid population compared with the female parent(*M. sacchariflorus*); (3) the DEGs between the F1 population and its male parent (*M. lutarioriparius*) annotated the functions of the carbohydrate metabolism pathway, and the fatty acid elongation pathway, which may have greater influence on yield and adaptability than other metabolic pathways.

## Figures and Tables

**Table 1:** Transcript length distribution of the assembled leaf reference transcriptome of *M. sacchariflorus*.

Length	Number	Frequency
100–500	377	4.65
500–1000	2271	27.98
1000–1500	2501	30.82
1500–2000	1549	19.09
2000–2500	757	9.33
2500–3000	311	3.83
3000–3500	184	2.27
3500–4000	73	0.90
4000–4500	38	0.47
4500–5000	23	0.28
≥5000	32	0.39
Total	8116	100

## Data Availability

The data are available from the corresponding author upon reasonable request.

## Supplementary Materials

The following supporting information can be downloaded at: https://www.mdpi.com/article/10.3390/plants11121568/s1, Table S1: The RNAseq data after being filtered and trimmed; Table S2: List of Gene Differential Expression Multiples of Three Groups of Differential Genes; Table S3: The Metabolism Pathways Annotations and Up-down Regulated Differential Genes in Three Groups.
