# Supplementary material for: Transcriptomic Characterization of Miscanthus sacchariflorus × M. lutarioriparius and Its Implications for Energy Crop Development in the Semiarid Mine Area
Source: Plants (Basel). 2022 Jun 14;11(12):1568. doi: 10.3390/plants11121568 (PMC9227993; doi:10.3390/plants11121568)
Supplement: Supplementary file 1 [file plants-11-01568-s001.zip › Table S1 The RNAseq data after being filtered and trimmed..pdf]

Table S1. The RNAseq data after being filtered and trimmed

Samples	Length of read (bp)	Number of reads sequenced	Number of reads with quality>20	Single-ended kept data volume (Gb)	Total G
1-1-1_clean_R1	91	44488440	43734360.94	1.99386066	3.979826846
1-1-1_clean_R2	91			1.985966186	
1-12-3_clean_R1	91	39420746	38870826.59	1.768353563	3.53724522
1-12-3_clean_R2	91			1.768891657	
1-2-2_clean_R1	91	53759084	53095159.31	2.421822543	4.831659497
1-2-2_clean_R2	91			2.409836955	
1-3-3_clean_R1	91	49616792	48788191.57	2.224603601	4.439725433
1-3-3_clean_R2	91			2.215121832	
1-5-1_clean_R1	91	54033274	53220073.23	2.42384892	4.843026664
1-5-1_clean_R2	91			2.419177744	
1-6-3_clean_R1	91	53094126	52284440.58	2.384135979	4.757884093
1-6-3_clean_R2	91			2.373748113	
1-7-1_clean_R1	91	45223950	44477754.83	2.028470531	4.047475689
1-7-1_clean_R2	91			2.019005158	
10-1-1_clean_R1	91	52775432	52010188.24	2.369104975	4.732927129
10-1-1_clean_R2	91			2.363822154	
10-11-1_clean_R1	91	48577526	47810001.09	2.179775604	4.350710099
10-11-1_clean_R2	91			2.170934495	
10-16-1_clean_R1	91	36791758	36306106.79	1.653267079	3.303855718
10-16-1_clean_R2	91			1.650588639	
10-2-1_clean_R1	91	59405176	58475485	2.66428353	5.321269135
10-2-1_clean_R2	91			2.656985604	
10-3-3_clean_R1	91	52834706	52044827.15	2.373688986	4.73607927
10-3-3_clean_R2	91			2.362390284	
10-4-1_clean_R1	91	81292020	79979153.88	3.645524378	7.278103003
10-4-1_clean_R2	91			3.632578624	
10-6-2_clean_R1	91	52599142	51728626.2	2.357362047	4.707304984
10-6-2_clean_R2	91			2.349942938	
11-1-2_clean_R1	91	45657504	44926983.94	2.04687841	4.088355538
11-1-2_clean_R2	91			2.041477128	
11-2-1_clean_R1	91	62345918	61370204.38	2.79560655	5.584688599
11-2-1_clean_R2	91			2.789082049	
11-3-1_clean_R1	91	55018164	54124118.84	2.467278561	4.925294814
11-3-1_clean_R2	91			2.458016253	
11-4-2_clean_R1	91	45801464	45052610.06	2.054582683	4.099787516
11-4-2_clean_R2	91			2.045204833	
11-7-1_clean_R1	91	53743888	52905483.35	2.412579255	4.814398985
11-7-1_clean_R2	91			2.401819729	
12-1-2_clean_R1	91	57055344	56156722.33	2.559414296	5.110261732

Samples	Length of read (bp)	Number of reads sequenced	Number of reads with quality>20	Single-ended kept data volume (Gb)	Total G
12-1-2_clean_R2	91			2.550847436	
12-2-3_clean_R1	91	49518306	48758200	2.225370003	4.4369962
12-2-3_clean_R2	91			2.211626197	
12-4-1_clean_R1	91	53836794	53072311.53	2.417729663	4.829580349
12-4-1_clean_R2	91			2.411850685	
12-8-3_clean_R1	91	69137188	68027536.13	3.099185072	6.190505788
12-8-3_clean_R2	91			3.091320716	
13-1-2_clean_R1	91	45128452	44415422.46	2.023981739	4.041803444
13-1-2_clean_R2	91			2.017821705	
13-11-1_clean_R1	91	52785984	51978358.44	2.369098305	4.730030618
13-11-1_clean_R2	91			2.360932313	
13-2-2_clean_R1	91	52614348	51909315.74	2.365464795	4.723747732
13-2-2_clean_R2	91			2.358282937	
13-4-2_clean_R1	91	53792858	53039757.99	2.419183169	4.826617977
13-4-2_clean_R2	91			2.407434808	
13-6-1_clean_R1	91	55830084	55073586.36	2.508261435	5.011696359
13-6-1_clean_R2	91			2.503434924	
14-1-1_clean_R1	91	54143698	53328835.35	2.431512262	4.852924016
14-1-1_clean_R2	91			2.421411755	
14-2-2_clean_R1	91	50777166	50132295.99	2.280788432	4.562038935
14-2-2_clean_R2	91			2.281250504	
14-3-2_clean_R1	91	49163634	48362266.77	2.204957029	4.400966276
14-3-2_clean_R2	91			2.196009247	
14-5-1_clean_R1	91	52659720	51861925.24	2.361514611	4.719435197
14-5-1_clean_R2	91			2.357920586	
14-7-1_clean_R1	91	44671582	43932267.32	2.003084905	3.997836326
14-7-1_clean_R2	91			1.994751421	
15-1-2_clean_R1	91	50911300	50094173.64	2.285655177	4.558569801
15-1-2_clean_R2	91			2.272914624	
15-2-1_clean_R1	91	47301510	46513939.86	2.121226756	4.232768527
15-2-1_clean_R2	91			2.111541771	
15-3-1_clean_R1	91	51289686	50458793.09	2.300775815	4.591750171
15-3-1_clean_R2	91			2.290974356	
15-4-1_clean_R1	91	48541086	47798407.38	2.177698741	4.349655072
15-4-1_clean_R2	91			2.171956331	
15-6-1_clean_R1	91	53753602	52874730.61	2.409102058	4.811600485
15-6-1_clean_R2	91			2.402498428	
15-7-2_clean_R1	91	47950364	47163978.03	2.150542658	4.291922001
15-7-2_clean_R2	91			2.141379343	
16-3-3_clean_R1	91	52032452	51106274.35	2.332911408	4.650670966
16-3-3_clean_R2	91			2.317759558	
16-4-3_clean_R1	91	48341364	47618660.61	2.167638847	4.333298115
16-4-3_clean_R2	91			2.165659268	

Samples	Length of read (bp)	Number of reads sequenced	Number of reads with quality>20	Single-ended kept data volume (Gb)	Total G
2-1-1_clean_R1	91	53870984	53046757.94	2.420000424	4.827254973
2-1-1_clean_R2	91			2.407254549	
2-12-1_clean_R1	91	45854618	45153042.34	2.056341173	4.108926853
2-12-1_clean_R2	91			2.05258568	
2-13-1_clean_R1	91	45077100	44344597.13	2.021678635	4.035358338
2-13-1_clean_R2	91			2.013679703	
2-14-1_clean_R1	91	46337476	45572907.65	2.077573173	4.147134596
2-14-1_clean_R2	91			2.069561423	
2-16-1_clean_R1	91	44815886	44116758.18	2.008943795	4.014624994
2-16-1_clean_R2	91			2.005681199	
2-2-1_clean_R1	91	50457256	49677691.39	2.262286393	4.520669917
2-2-1_clean_R2	91			2.258383524	
2-3-2_clean_R1	91	52656540	51771910.13	2.360413656	4.711243822
2-3-2_clean_R2	91			2.350830166	
2-4-1_clean_R1	91	60477020	59509387.68	2.711254355	5.415354279
2-4-1_clean_R2	91			2.704099924	
2-6-2_clean_R1	91	51617110	50884147.04	2.313584685	4.63045738
2-6-2_clean_R2	91			2.316872695	
2-7-2_clean_R1	91	51821378	51044057.33	2.32321197	4.645009217
2-7-2_clean_R2	91			2.321797247	
2-8-3_clean_R1	91	64157416	63169391.79	2.878294154	5.748414653
2-8-3_clean_R2	91			2.870120499	
3-1-2_clean_R1	91	58343810	57465735.66	2.621991242	5.229381945
3-1-2_clean_R2	91			2.607390703	
3-10-3_clean_R1	91	59499040	58463756.7	2.669305432	5.32020186
3-10-3_clean_R2	91			2.650896429	
3-11-2_clean_R1	91	52398068	51530879.97	2.349304057	4.689310078
3-11-2_clean_R2	91			2.34000602	
3-13-1_clean_R1	91	51795180	50958687.84	2.321801816	4.637240594
3-13-1_clean_R2	91			2.315438778	
3-14-2_clean_R1	91	53487890	52640106.94	2.400113949	4.790249732
3-14-2_clean_R2	91			2.390135783	
3-15-1_clean_R1	91	56649612	55646913.87	2.546884414	5.063869162
3-15-1_clean_R2	91			2.516984748	
3-3-2_clean_R1	91	54891848	53961431.18	2.462612977	4.910490237
3-3-2_clean_R2	91			2.44787726	
3-6-1_clean_R1	91	46114594	45316811.52	2.069678316	4.123829849
3-6-1_clean_R2	91			2.054151532	
3-7-1_clean_R1	91	47519114	46592491.28	2.128174408	4.239916706
3-7-1_clean_R2	91			2.111742298	
3-8-2_clean_R1	91	53540982	52687003.34	2.402496298	4.794517304
3-8-2_clean_R2	91			2.392021005	
4-1-1_clean_R1	91	50852730	50028915.77	2.280711886	4.552631335

Samples	Length of read (bp)	Number of reads sequenced	Number of reads with quality>20	Single-ended kept data volume (Gb)	Total G
4-1-1_clean_R2	91				
4-11-2_clean_R1	91	47547728	46794096.51	2.131402984	4.258262783
4-11-2_clean_R2	91			2.126859799	
4-12-3_clean_R1	91	47858396	47083089.98	2.145764686	4.284561189
4-12-3_clean_R2	91			2.138796503	
4-15-3_clean_R1	91	53290822	52443497.93	2.390058728	4.772358312
4-15-3_clean_R2	91			2.382299584	
4-2-2_clean_R1	91	51174676	50309823.98	2.29491811	4.578193982
4-2-2_clean_R2	91			2.283275871	
4-3-1_clean_R1	91	53280282	52502389.88	2.392010268	4.777717479
4-3-1_clean_R2	91			2.385707211	
4-4-1_clean_R1	91	57682674	56852043.49	2.587555347	5.173535958
4-4-1_clean_R2	91			2.58598061	
4-7-2_clean_R1	91	51224998	50413081.78	2.303234708	4.587590442
4-7-2_clean_R2	91			2.284355735	
4-8-1_clean_R1	91	56210042	55437153.92	2.521239603	5.044781007
4-8-1_clean_R2	91			2.523541404	
4-9-1_clean_R1	91	51765188	51043063.63	2.323283756	4.64491879
4-9-1_clean_R2	91			2.321635034	
5-11-1_clean_R1	91	53008724	52285154.92	2.382954179	4.757949097
5-11-1_clean_R2	91			2.374994919	
5-12-1_clean_R1	91	58720692	57836945.59	2.633317689	5.263162048
5-12-1_clean_R2	91			2.62984436	
5-15-1_clean_R1	91	54372922	53500236.6	2.440569384	4.868521531
5-15-1_clean_R2	91			2.427952147	
5-16-1_clean_R1	91	47355246	46633078.5	2.124498401	4.243610143
5-16-1_clean_R2	91			2.119111742	
5-2-2_clean_R1	91	52315292	51486094.62	2.346068807	4.685234611
5-2-2_clean_R2	91			2.339165804	
5-7-1_clean_R1	91	48898642	48177387.03	2.192849821	4.38414222
5-7-1_clean_R2	91			2.191292399	
5-8-3_clean_R1	91	51466388	50707258.78	2.31291749	4.614360549
5-8-3_clean_R2	91			2.301443059	
5-9-1_clean_R1	91	47441438	46798606.52	2.126854223	4.258673193
5-9-1_clean_R2	91			2.13181897	
6-13-1_clean_R1	91	59205960	58237942.55	2.654810048	5.299652772
6-13-1_clean_R2	91			2.644842725	
6-16-1_clean_R1	91	50613144	49793211.07	2.271578638	4.531182207
6-16-1_clean_R2	91			2.259603569	
6-3-1_clean_R1	91	53977590	53192216.07	2.422087816	4.840491662
6-3-1_clean_R2	91			2.418403846	
6-7-2_clean_R1	91	46811626	46032212.43	2.099896984	4.188931331
6-7-2_clean_R2	91			2.089034347	

Samples	Length of read (bp)	Number of reads sequenced	Number of reads with quality>20	Single-ended kept data volume (Gb)	Total G
6-8-1_clean_R1	91	48768920	47954479.04	2.187920058	4.363857592
6-8-1_clean_R2	91			2.175937534	
6-9-1_clean_R1	91	51424944	50679282.31	2.311756933	4.61181469
6-9-1_clean_R2	91			2.300057758	
7-1-2_clean_R1	91	48600802	47806178.89	2.178608711	4.350362279
7-1-2_clean_R2	91			2.171753568	
7-11-1_clean_R1	91	47116372	46357798.41	2.114210556	4.218559655
7-11-1_clean_R2	91			2.104349099	
7-16-1_clean_R1	91	56069298	55287131.29	2.519773876	5.031128948
7-16-1_clean_R2	91			2.511355071	
7-3-1_clean_R1	91	55733924	54903488.53	2.505462819	4.996217456
7-3-1_clean_R2	91			2.490754637	
7-4_clean_R1	91	48767910	47977869.86	2.187874746	4.365986157
7-4_clean_R2	91			2.178111411	
7-5-1_clean_R1	91	52993386	52166689.18	2.377924516	4.747168715
7-5-1_clean_R2	91			2.369244199	
7-7-1_clean_R1	91	46613738	45823635.14	2.090807943	4.169950798
7-7-1_clean_R2	91			2.079142855	
8-1-1_clean_R1	91	54155118	53291343.87	2.430300276	4.849512292
8-1-1_clean_R2	91			2.419212016	
8-11-2_clean_R1	91	50050552	49212205.25	2.244506994	4.478310678
8-11-2_clean_R2	91			2.233803684	
8-12-3_clean_R1	91	57584698	56620154.31	2.582636275	5.152434042
8-12-3_clean_R2	91			2.569797767	
8-16-1_clean_R1	91	56073332	55209802.69	2.51816923	5.024092045
8-16-1_clean_R2	91			2.505922814	
8-2-2_clean_R1	91	56328498	55477937.68	2.530397236	5.048492329
8-2-2_clean_R2	91			2.518095092	
8-3-1_clean_R1	91	52879772	52017831.72	2.368254961	4.733622686
8-3-1_clean_R2	91			2.365367725	
8-7-1_clean_R1	91	46245356	45385192.38	2.073863741	4.130052506
8-7-1_clean_R2	91			2.056188766	
9-1-2_clean_R1	91	51036932	50087645.06	2.282935557	4.557975701
9-1-2_clean_R2	91			2.275040144	
9-13-1_clean_R1	91	45771570	45075842.14	2.052825163	4.101901634
9-13-1_clean_R2	91			2.049076471	
9-16-2_clean_R1	91	47931700	47064136.23	2.150141768	4.282836397
9-16-2_clean_R2	91			2.132694629	
9-2-1_clean_R1	91	48360750	47623248.56	2.169168244	4.333715619
9-2-1_clean_R2	91			2.164547375	
9-3-1_clean_R1	91	44194388	43345855.75	1.982089575	3.944472873
9-3-1_clean_R2	91			1.962383298	
9-4-3_clean_R1	91	48250214	47478210.58	2.163990735	4.320517162

**Samples**	**Length of read (bp)**	**Number of reads sequenced**	**Number of reads with quality>20**	**Single-ended kept data volume (Gb)**	**Total G**
9-4-3_clean_R2	91				
9-5-3_clean_R1	91	57158366	56255263.82	2.563775633	5.119229007
9-5-3_clean_R2	91			2.555453375	
9-6-1_clean_R1	91	53443792	52615413.22	2.398135179	4.788002603
9-6-1_clean_R2	91			2.389867424	
9-7-1_clean_R1	91	50975670	50307888.72	2.294343541	4.578017874
9-7-1_clean_R2	91			2.283674333	
I-6-10-2_clean_R1	91	48985258	48228435.76	2.19762563	4.388787655
I-6-10-2_clean_R2	91			2.191162025	
QBD-2_clean_R1	91	31192674	30709187.55	1.399396934	2.794536067
QBD-2_clean_R2	91			1.395139134	
QBND-2_clean_R1	91	53426130	52675492.87	2.398558092	4.793469851
QBND-2_clean_R2	91			2.394911759	
