# Supplementary material for: Transcriptomic Characterization of Miscanthus sacchariflorus × M. lutarioriparius and Its Implications for Energy Crop Development in the Semiarid Mine Area
Source: Plants (Basel). 2022 Jun 14;11(12):1568. doi: 10.3390/plants11121568 (PMC9227993; doi:10.3390/plants11121568)
Supplement: Supplementary file 1 [file plants-11-01568-s001.zip › Table S2 List of Gene Differential Expression Multiples of Three Groups of Differential Genes.pdf]

Table S2. List of Gene Differential Expression Multiples of Three Groups of Differential Genes

Genes ID	DEG1（F1 VS *M.sacchariflorus*）		DEG2（F1 VS *M.lutarioriparius*）		DEG3（*M.sacchariflorus* VS *M.lutarioriparius*		notes
	log2(FoldChange)	Regulation	log2(FoldChange)	Regulation	log2(FoldChange)	Regulation	
K41_C316020	-1.310712759	down	6.210400498	up	7.894091613	up	Shared gene
MluLR5042	-1.134759322	down	1.207996123	up	2.377303044	up	Shared gene
K41_C314568	-1.106776896	down	3.795597058	up	5.116595396	up	Shared gene
MluLR17506	-3.018267179	down	-1.386026048	down	1.618657177	up	Shared gene
MluLR7864	2.653612163	up	-1.59550086	down	-4.534463024	down	Shared gene
MluLR3275	4.901128752	up	-1.033135479	down	-6.716931891	down	Shared gene
K41_C234516	-2.467297676	down	——	——	3.906518874	up	Special gene in female
K41_C293894	-1.999383638	down	——	——	5.846984104	up	Special gene in female
MluLR9293	-3.582257016	down	——	——	4.330865357	up	Special gene in female
MluLR14171	-2.660586136	down	——	——	2.920393234	up	Special gene in female
K41_C312250	-2.640868129	down	——	——	2.947430247	up	Special gene in female
K41_C315280	-1.731277353	down	——	——	3.031240453	up	Special gene in female
MluLR10849	-1.719890672	down	——	——	2.533910335	up	Special gene in female

Genes ID	DEG1（F1 VS *M.sacchariflorus*）		DEG2（F1 VS *M.lutarioriparius*）		DEG3（*M.sacchariflorus* VS *M.lutarioriparius*）		notes
	log2(FoldChange)	Regulation	log2(FoldChange)	Regulation	log2(FoldChange)	Regulation	
MluLR7765	-1.327309567	down	——	——	1.983842989	up	Special gene in female
MluLR9600	1.531903716	up	——	——	-2.232163456	down	Special gene in female
MluLR1095	1.962500737	up	——	——	-2.302934658	down	Special gene in female
MluLR1136	2.202262544	up	——	——	-2.677583045	down	Special gene in female
MluLR8565	2.620413984	up	——	——	-3.26452968	down	Special gene in female
MluLR522	2.768744757	up	——	——	-3.114343886	down	Special gene in female
MluLR866	2.835889981	up	——	——	-3.392799407	down	Special gene in female
MluLR7052	2.868002423	up	——	——	-3.836759967	down	Special gene in female
MluLR5589	3.004559452	up	——	——	-4.409667274	down	Special gene in female
MluLR11938	3.009790294	up	——	——	-3.722479591	down	Special gene in female
MluLR12032	3.034736934	up	——	——	-3.700377543	down	Special gene in female

Genes ID	DEG1（F1 VS *M.sacchariflorus*）		DEG2（F1 VS *M.lutarioriparius*）		DEG3（*M.sacchariflorus* VS *M.lutarioriparius*		notes
	log2(FoldChange)	Regulation	log2(FoldChange)	Regulation	log2(FoldChange)	Regulation	
MluLR173	3.085295742	up	——	——	-4.484100601	down	Special gene in female
MluLR547	3.184687715	up	——	——	-4.523585149	down	Special gene in female
MluLR14284	3.262087745	up	——	——	-3.807535039	down	Special gene in female
MluLR4295	3.694526265	up	——	——	-4.918811601	down	Special gene in female
MluLR4430	3.697432047	up	——	——	-5.014681131	down	Special gene in female
MluLR10658	3.96448026	up	——	——	-4.574209078	down	Special gene in female
MluLR16622	4.187043391	up	——	——	-5.094321356	down	Special gene in female
MluLR12926	4.34834363	up	——	——	-4.723583903	down	Special gene in female
MluLR7800	4.628757594	up	——	——	-6.455196979	down	Special gene in female
MluLR13074	5.124462195	up	——	——	-5.613101069	down	Special gene in female
MluLR9925	5.181407175	up	——	——	-6.151757592	down	Special gene in female

Genes ID	DEG1（F1 VS *M.sacchariflorus*）		DEG2（F1 VS *M.lutarioriparius*）		DEG3（*M.sacchariflorus* VS *M.lutarioriparius*		notes
	log2(FoldChange)	Regulation	log2(FoldChange)	Regulation	log2(FoldChange)	Regulation	
MluLR8018	5.315341345	up	——	——	-6.879088338	down	Special gene in female
MluLR863	5.86104175	up	——	——	-7.409988854	down	Special gene in female
MluLR10478	6.176455702	up	——	——	-7.195964028	down	Special gene in female
MluLR11561	——	——	-2.519564614	down	-4.308497635	down	Special gene in male
MluLR8367	——	——	-1.454118256	down	-4.451498976	down	Special gene in male
MluLR8818	——	——	-1.417877597	down	-3.509151551	down	Special gene in male
MluLR13134	——	——	-1.235348518	down	-2.408920686	down	Special gene in male
MluLR7989	——	——	-1.224506666	down	-1.947200315	down	Special gene in male
MluLR5039	——	——	-1.179279053	down	-1.954943223	down	Special gene in male
MluLR12920	——	——	-1.171837288	down	-2.914360594	down	Special gene in male
MluLR13656	——	——	-1.142505676	down	-2.944666935	down	Special gene in male

Genes ID	DEG1（F1 VS *M.sacchariflorus*）		DEG2（F1 VS *M.lutarioriparius*）		DEG3（*M.sacchariflorus* VS *M.lutarioriparius*		notes
	log2(FoldChange)	Regulation	log2(FoldChange)	Regulation	log2(FoldChange)	Regulation	
K41_scaffold13579	——	——	1.310948261	up	1.630580575	up	Special gene in male
Cap3i95_Contig32036	——	——	1.361092931	up	1.587620729	up	Special gene in male
Cap3i94_Contig4783	——	——	1.411582504	up	2.395017543	up	Special gene in male
K41_scaffold1343	——	——	1.422305257	up	2.507670222	up	Special gene in male
K41_scaffold1865	——	——	1.484796406	up	2.35984742	up	Special gene in male
Cap3i94_Contig4598	——	——	1.680471097	up	2.3759353	up	Special gene in male
K41_C316852	——	——	1.704902246	up	1.274974515	up	Special gene in male
MluLR7657	——	——	1.764699655	up	2.323282252	up	Special gene in male
Cap3i94_Contig3416	——	——	1.845008878	up	2.981480289	up	Special gene in male
MluLR15067	——	——	1.902757151	up	3.162190831	up	Special gene in male
K41_scaffold14908	——	——	1.961047524	up	2.524477436	up	Special gene in male

Genes ID	DEG1（F1 VS *M.sacchariflorus*）		DEG2（F1 VS *M.lutarioriparius*）		DEG3（*M.sacchariflorus* VS *M.lutarioriparius*		notes
	log2(FoldChange)	Regulation	log2(FoldChange)	Regulation	log2(FoldChange)	Regulation	
Cap3i97_Contig2585	——	——	1.970469177	up	2.497857506	up	Special gene in male
Cap3i94_Contig28339	——	——	2.014524382	up	2.277640184	up	Special gene in male
K61_scaffold3236	——	——	2.113558929	up	2.958379626	up	Special gene in male
MluLR1093	——	——	2.272019255	up	2.933764132	up	Special gene in male
K51_scaffold12551	——	——	2.593865838	up	3.435471282	up	Special gene in male
K41_scaffold13483	——	——	2.611655173	up	3.302503281	up	Special gene in male
K51_scaffold12750	——	——	2.622661891	up	3.318228227	up	Special gene in male
K51_C245430	——	——	2.631147036	up	3.975537724	up	Special gene in male
MluLR8595	——	——	2.838171278	up	2.646369957	up	Special gene in male
K41_C317200	——	——	2.885646422	up	3.772971622	up	Special gene in male
K41_scaffold2760	——	——	2.907625187	up	2.927164006	up	Special gene in male

Genes ID	DEG1（F1 VS *M.sacchariflorus*）		DEG2（F1 VS *M.lutarioriparius*）		DEG3（*M.sacchariflorus* VS *M.lutarioriparius*		notes
	log2(FoldChange)	Regulation	log2(FoldChange)	Regulation	log2(FoldChange)	Regulation	
MluLR3385	——	——	2.915141257	up	3.795654281	up	Special gene in male
MluLR16240	——	——	3.132804182	up	4.081445375	up	Special gene in male
K41_scaffold9209	——	——	3.356017732	up	3.983372537	up	Special gene in male
MluLR7808	——	——	3.418072787	up	4.238508046	up	Special gene in male
MluLR1137	——	——	3.803629542	up	4.946398817	up	Special gene in male
Cap3i94_Contig5243	——	——	3.817698096	up	5.461881175	up	Special gene in male
K41_scaffold8733	——	——	3.942657081	up	4.060251694	up	Special gene in male
MluLR9531	——	——	4.001188839	up	4.622841112	up	Special gene in male
MluLR4601	——	——	4.381250266	up	6.943004662	up	Special gene in male
Cap3i94_Contig3566	——	——	4.755337989	up	6.004998228	up	Special gene in male
K41_scaffold16362	——	——	4.793801101	up	5.980813882	up	Special gene in male

Genes ID	DEG1（F1 VS *M.sacchariflorus*）		DEG2（F1 VS *M.lutarioriparius*）		DEG3（*M.sacchariflorus* VS *M.lutarioriparius*		notes
	log2(FoldChange)	Regulation	log2(FoldChange)	Regulation	log2(FoldChange)	Regulation	
Cap3i94_Contig5885	——	——	5.073914834	up	5.96013965	up	Special gene in male
MluLR7772	——	——	5.410270836	up	6.700246247	up	Special gene in male
K61_C140346	——	——	5.888314699	up	7.135694261	up	Special gene in male
Cap3i94_Contig5889	——	——	5.98504523	up	7.654340148	up	Special gene in male
K41_scaffold15739	——	——	6.055356158	up	7.744241047	up	Special gene in male
MluLR7047	——	——	6.357827388	up	7.117399267	up	Special gene in male
Cap3i94_Contig5645	——	——	6.885703871	up	7.734530695	up	Special gene in male
K61_C145926	——	——	7.171568049	up	6.959085157	up	Special gene in male
