# Supplementary material for: Transcriptomic Characterization of Miscanthus sacchariflorus × M. lutarioriparius and Its Implications for Energy Crop Development in the Semiarid Mine Area
Source: Plants (Basel). 2022 Jun 14;11(12):1568. doi: 10.3390/plants11121568 (PMC9227993; doi:10.3390/plants11121568)
Supplement: Supplementary file 1 [file plants-11-01568-s001.zip › Table S3 The Enrichment and Metabolism Pathways and Up.pdf]

Table S3. The Metabolism Pathways Annotations and Up-down Regulated Differential Genes in Three Groups

Pathway of A class	Pathway of B class	pathway ID	DEG3			DEG1			DEG2		
			Pathway of C class	The number of upregulated genes	The number of downregulated genes	Pathway of C class	The number of upregulated genes	The number of downregulated genes	Pathway of C class	The number of upregulated genes	The number of downregulated genes
1. Metabolism	1.0 Global and overview maps	ath01230	Biosynthesis of amino acids	16	16	——	—	—	——	—	—
		ath01110	Biosynthesis of secondary metabolites	90	74	Biosynthesis of secondary metabolites	3	1	Biosynthesis of secondary metabolites	2	0
		ath01200	Carbon metabolism	10	18	——	——	——	——	——	——
		ath01212	Fatty acid metabolism	9	2	Fatty acid metabolism	1	1	Fatty acid metabolism	1	1
		ath01100	Metabolic pathways	144	117	Metabolic pathways	4	2	Metabolic pathways	1	1
	1.1 Carbohy-drate metaboli-sm	ath00520	Amino sugar and nucleotide sugar metabolism	6	5	——	——	——	——	——	——
		ath00010	Glycolysis / Gluconeogenesis	4	7	——	——	——	——	——	——
		ath00630	Glyoxylate and dicarboxylate metabolism-	3	7	——	——	——	——	——	——

	ath00640	Propanoate metabolism-	7	2	——	——	——	——	——	——
	ath00230	Purine metabolism-	5	6	——	——	——	——	——	——
	ath00620	Pyruvate metabolism	4	4	——	——	——	——	——	——
	ath00500	Starch and sucrose metabolism	6	17	Starch and sucrose metabolism	2	0	Starch and sucrose metabolism	2	0
1.2 Energy metaboli -sm	ath00920	Sulfur metabolism	3	4	——	——	——	——	——	——
1.3 Lipid metaboli -sm	ath00592	alpha-Linolenic acid metabolism	3	4	——	——	——	——	——	——
	ath01040	Biosynthesis of unsaturated fatty acids	3	1	——	——	——	——	——	——
	ath00061	Fatty acid biosynthesis	6	0	——	——	——	——	——	——
	ath00071	Fatty acid degradation	6	1	——	——	——	——	——	——
	ath00062	Fatty acid elongation	2	3	Fatty acid elongation	1	1	Fatty acid elongation	4	2

	ath00100	Steroid biosynthesis	2	3	Steroid biosynthesis	0	1			
	ath00250	Arginine and proline metabolism	5	3	——	——	——	——	——	——
	ath00330	Alanine, aspartate and glutamate metabolism	4	2	——	——	——	——	——	——
	ath00270	Cysteine and methionine metabolism	9	5	——	——	——	——	——	——
1.5 Amino acid metaboli-sm					——	——	——	**Histidine metabolism**	0	1
	ath00260	Glycine, serine and threonine metabolism	4	9	——	——	——	——	——	——
	ath00360	Phenylalanine metabolism	12	2	——	——	——	——	——	——
	ath00380	Tryptophan metabolism	8	1	——	——	——	——	——	——
	ath00350	Tyrosine metabolism	5	2	——	——	——	——	——	——
	ath00280	Valine, leucine and isoleucine degradation	4	2	——	——	——	——	——	——

1.6 Metaboli-sm of other amino acids	ath00410	beta-Alanine metabolism	4	5	——	——	——	——	——	——
	ath00460	Cyanoamino acid metabolism	5	7	——	——	——	Cyanoamino acid metabolism	5	3
	ath00480	Glutathione metabolism	14	6	——	——	——	——	——	——
	ath00450	Selenocompound metabolism	3	1	——	——	——	——	——	——
1.8Meta-bolism of cofactors and vitamins	ath00760	Nicotinate and nicotinamide metabolism	1	2	——	——	——	——	——	——
	——	——	——	——	——	——	——	**One carbon pool by folate**	2	0
	ath00130	Ubiquinone and other terpenoid-quinone biosynthesis	12	2	——	——	——	——	——	——
1.9Metabolism of terpenoids and polyketides	ath00906	Carotenoid biosynthesis	2	3	——	——	——	——	——	——
	ath00902	Monoterpenoid biosynthesis	3	0	——	——	——	Monoterpenoid biosynthesis	1	0
1.10 Biosynthes	ath00941	Flavonoid biosynthesis	17	3	——	——	——	——	——	——

	is of other secondary metabolites	ath00950	Isoquinoline alkaloid biosynthesis	4	2	——	——	——	——	——	——
		ath00940	Phenylpropanoid biosynthesis	28	5	——	——	——	Phenylpropanoid biosynthesis	2	0
		ath00945	Stilbenoid, diarylheptanoid and gingerol biosynthesis	4	1	——	——	——	——	——	——
		ath00960	Tropane, piperidine and pyridine alkaloid biosynthesis	4	2	——	——	——	——	——	——
2. Genetic Information Processing	2.1Transcription	ath03020	RNA polymerase	3	7	RNA polymerase	1	0	——	——	——
	2.4 Replication and repair	ath03030	DNA replication	3	3	——	——	——	——	——	——
		ath03430	Mismatch repair	2	3	——	——	——	——	——	——
3.Environmental Information Processing	3.2 Signal transduc-tion	ath04016	MAPK signaling pathway - plant	9	4	——	——	——	——	——	——
		ath04075	Plant hormone signal transduction	13	9	——	——	——	——	——	——

4.Cellular Processes	4.1 Transport and catabolism	ath04146	Peroxisome	12	5	——	——	——	——	——	——
		ath04145	Phagosome	2	7	——	——	——	——	——	——
5. Organismal Systems	5.10Environmental adaptation	ath04712	Circadian rhythm - plant	6	2	——	——	——	Circadian rhythm-plant	1	0
		ath04626	Plant-pathogen interaction	13	4	——	——	——	——	——	——
